# Importance of the 1921 Census

**Published:** 1921-01-29

**Authors:** 


THE PUBLIC WELL-BEING—(*continued*).

### IMPORTANCE OF THE 1921 CENSUS.

It usually happens in the months immediately preceding the taking of the Census that the authorities make a prolonged effort through the press to stimulate public interest in the question, because the co-operation of the public not only greatly simplifies the work of the officials, but tends to make the results more accurate and more valuable to the community. It is understood that the new Registrar-General regards the 1921 Census as the most valuable inquiry of the kind ever undertaken in this country. This opinion is based on the important changes brought about by the war, which has restricted the movements of the people, upset the returns of births and deaths, changed the whole face of industry, caused a redistribution of the population in many areas, created new problems of housing and employment, and altered most of our habits and customs.

### A Modification.

In this connection it is interesting to observe that the Registrar-General has already considerably modified the preposterous official estimate for 1919, which we subjected to analysis last week, that only 140,000 new houses are required to meet the existing shortage. It seems evident that the Prime Minister's public estimate, in which he placed the figure at 500,000 (less slum areas—awaiting demolition—that are not fit for human habitation), has been brought to the notice of the new chief of the Department, for he now says that the report did not suggest that the requirements could be met by the erection of 140,000 houses. The plain man will wonder what it did suggest, if not that, for the statement appeared to be categorical. But the Ministry of Health, at any rate, will be satisfied with the disclaimer, however disingenuous it may seem. The "explanation" now is that the report in question provided population statistics bearing upon housing requirements "arising out of that cause alone," a cryptic phrase that may be interpreted at will. It was the annual report for 1919, we are reminded, and the calculations referred to were based upon figures for the population of the mid-year—June 30. Therefore the estimate should not have been applied to conditions in 1921. Since June 1919 the civil population has been increased by demobilisation and by births, and "if the calculations could now be made in respect of the present date, the results would be different." This "explanation" or withdrawal is satisfactory so far as it goes, but it really "explains" nothing that was not known before. The Prime Minister's figure was one generally accepted in 1919, and all that we can now infer is that in all probability it will be found to be short of the truth when the details of the 1921 Census are collected and classified.

### A Difference in Forms.

It may be useful to point out with regard to the forthcoming Census inquiry that the householders' forms, of which eleven millions are to be distributed, will differ in several respects from the forms issued in 1911. For instance, the "fertility inquiry" (the record of the number of years of marriage and the number of living children born in those years) has been discarded, as well as the columns relating to blindness, deafness, and imbecility. These details, it was found, were too imperfectly supplied to be of service. Instead, new inquiries have been introduced with reference to a man's place of work and the number of his dependent children. The place of work in relation to the place of residence, it is explained, has an important bearing upon such factors as transport and housing and continuation schools. Obviously, to decide upon a scientific and economic basis where to build houses in large groups must depend upon precise knowledge of the exact locality in which the people who are to occupy them will work. A man will also be tabulated both in regard to the occupation and the industry which employs him. Such descriptions as "artisan," "factory hand," "operative," and "carman" are of limited value, seeing that a carman, for example, may be employed by a brewery firm or by a textile manufacturer; while under the new classification this distinction is made—that the occupation concerns his health and the industry concerns his prosperity.

### Women at Home.

More careful methods are also to be introduced for tabulating women at home. Formerly a woman working about the house was included in the "no occupation" class; and there was nothing to distinguish her from other women who did no work at all. Her occupation will now be entered as "home duties." Also in cases in which it might be undesirable for the head of the household to fill up all the particulars required in respect of certain individuals in the house, especially in the case of domestic servants, arrangements are to be made in which it will be possible for a person who does not desire to disclose the required information to the head of the house to go to the local registrar and receive from him a form which he or she will be able to fill up and present to the enumerator when the latter delivers the household schedules, upon which a separate schedule will be provided.

### Counting the Transfer.

The census extends to a three-mile radius round the coast. The Admiralty will take this in hand on King's ships, the Customs and coastguards will be responsible for the mercantile marine, and the local enumerators will visit the fringe of coasting vessels. These enumerators will also have to get the particulars from gipsies and caravan and tent dwellers, while the local police will count the tramps. It is going to be an exhaustive census, and the statistical material which it will make available to the authorities should have a distinct bearing on the future well-being of the nation.

